# A review of 17 cases of mesenteric panniculitis in Zhengzhou Ninth People’s Hospital in China

**DOI:** 10.1186/s12876-024-03136-8

**Published:** 2024-01-24

**Authors:** Hongyan Wang, Zhenyu Zhao, Qiucai Cao, Jing Ning

**Affiliations:** 1 Geriatric Center, Zhengzhou Ninth People’s Hospital, 25 Sha Kou Road, 450008 Zhengzhou, Henan China; 2 Radiology, Zhengzhou Ninth People’s Hospital, 25 Sha Kou Road, 450008 Zhengzhou, Henan China

**Keywords:** Mesenteric panniculitis, Abdominal pain, CT, Chronic

## Abstract

**Purpose:**

Mesenteric panniculitis (MP) represents the uncommon, benign and chronic inflammatory disorder affecting the mesenteric adipose tissues. Its etiology, diagnosis and treatment remain unnoticed. Our report focused on shedding more lights on this condition.

**Patients and methods:**

Seventeen MP patients were identified by searching the electronic medical record system in the Zhengzhou Ninth People’s Hospital using the search terms “Mesenteric panniculitis” from October 2015 to March 2023. All cases were diagnosed with MP through computed tomography (CT). Their clinical features and treatments were analyzed.

**Results:**

There were altogether 17 cases enrolled for this analysis. The male to female ratio was 8:9, and the median age at diagnosis was 64 (range: 37–96) years. There were 15 patients (88.2%) showing abdominal pain to varying degrees. The proportions of symptoms of nausea, vomiting and fever were 23.5%, 23.5% and 41.2%, respectively. Neoplastic disease was present in 3 patients (17.6%). Meanwhile, 9 patients (52.9%) had gallstones, 3 (17.6%) had cholecystitis and 1 (5.9%) had gallbladder polyps. Six patients (35.3%) received antibiotics treatment only and 1 (5.9%) received oral antibiotics and prednisone. One patient (5.9%) received antibiotics followed by prednisone treatment, because the symptoms were significantly relieved after antibiotic treatment, while the disease recurred soon after, and the symptoms improved again after prednisone treatment. The abdominal pain in 9 patients (52.9%) was relieved spontaneously. Two patients (11.8%) died, including one due to respiratory failure caused by pneumonia and the other one because of pancreatic cancer with lung and liver metastases.

**Conclusion:**

MP is a poorly understood chronic inflammatory disease. Patients often have abdominal pain as the main symptom, accompanied by comorbidities in the gallbladder, and the prognosis is usually good after correct diagnosis and treatment, Therefore, the present report aims to promote the awareness among clinicians of patients with non-classic abdominal symptoms, so as to avoid misdiagnosis or missed diagnosis.

## Introduction

Mesenteric panniculitis (MP) accounts for the disorder characterized by chronic non-specific unexplained inflammation. It was first described as “sclerosing mesenteritis” by Jura in 1924 and further labeled as “MP” by Odgen later in 1965 [1]. In an autopsy study of the mesenterium of all adults during a period of 6 months, 9 out of 712 autopsies (1.26%) showed mesenteritis [2]. Combined with most literature reports, the incidence of mesenteritis is about 0.16-3.4% [3, 4]. Most studies have indicated that MP is more commonly found among men, and the male-to-female ratio is 2–3:1 [5]. At present, the precise pathophysiological mechanism of MP is still unclear, but it is probably associated with various conditions like abdominal trauma/surgery, cancer, inflammatory disease, mesenteric ischemia, obesity [6], acute pancreatitis [7], autoimmune disease [8, 9], Coronavirus Disease (COVID-19) [10] and so on. This work focused on investigating the clinical characteristics and treatment in 17 MP cases.

## Materials and methods

The present retrospective study was conducted from October 2015 to March 2023. In total, 17 patients with MP were identified by searching the electronic medical record system in Zhengzhou Ninth People’s Hospital using the search term “Mesenteric panniculitis”.

These cases were all diagnosed through computed tomography (CT) by an independent radiologist to review the images. The initial of the radiologist was “ZZ”. In this work, we eliminated cases known to display the CT findings close to MP (namely, recent mesenteric trauma, mesenteric edema, mesenteric lymphoma or portal hypertension).

## CT analysis

**Fig. 1:**
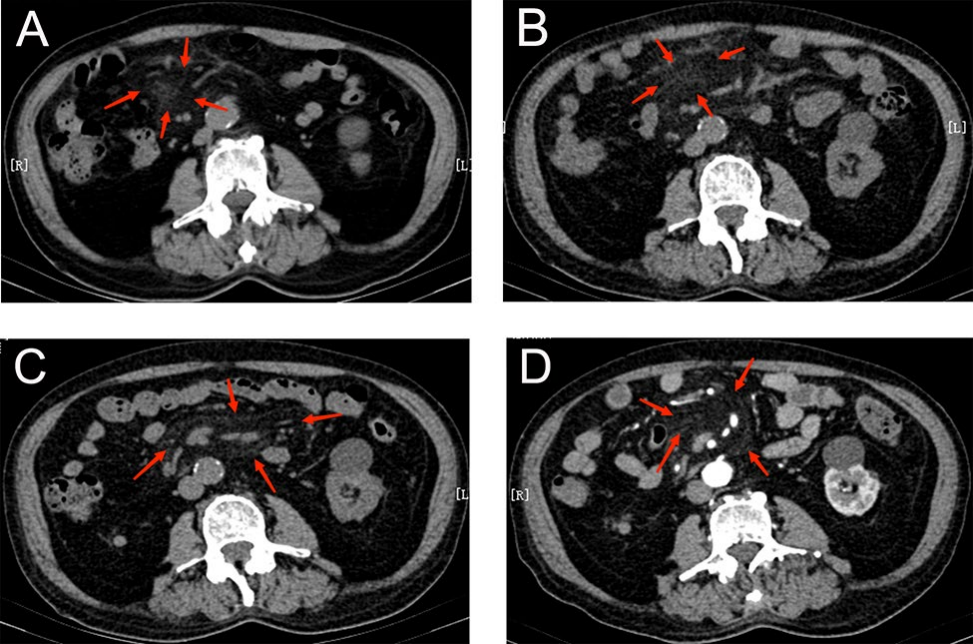
A 96-year-old man with abdominal pain on the right side. Plain abdominal CT revealed hyper-attenuation of mesenteric fat (**A**). After antibiotic treatment, the abdominal pain was mitigated but soon worsened (**B**). The abdominal pain was gradually controlled following the initiation of prednisone therapy (40 mg daily), repeat CT revealed less panniculitis (**C**), and contrast-enhanced CT [iodinated contrast agent, Iohexl Injection, GE HEALTHCARE (Shanghai) Co., Ltd., China] revealed hyper-attenuation of mesenteric root, accompanied by a fat halo sign that surrounded the vessels, which suggested MP (**D**)

CT findings evaluated in the present work were depicted previously for MP patients in literature reports, including hyper-attenuation of mesenteric fat called the “misty mesentery”, and fat halo sign around nodules and/or vessels [4, 6, 11, 12]. In the present work, MP was confirmed in the presence of hyper-attenuation of mesenteric fat (Fig. 1), which has been most extensively mentioned for MP in previous studies [4, 11].

## Results

In our study, clinical characteristics, treatment as well as long-term outcomes including the risk of recurrence and the impact on quality of life of those seventeen MP patients were analyzed (Table 1). According to our results, the male-to-female ratio was 8:9, and the median age at diagnosis was 64 (range, 37–96) years. There were 15 patients (88.2%) showing abdominal pain to varying degrees. The proportions of symptoms of nausea, vomiting and fever were 23.5%, 23.5% and 41.2%, respectively. Neoplastic disease was present in 3 patients (17.6%). Meanwhile, 9 patients (52.9%) had gallstones, 3 (17.6%) had cholecystitis and 1 (5.9%) had gallbladder polyps. Six patients (35.3%) received antibiotics alone. One (5.9%) took oral antibiotics and prednisone. One (5.9%) received antibiotics followed by prednisone, because the symptoms were significantly relieved after antibiotic treatment, but aggravated soon thereafter, and the symptoms improved again after prednisone treatment. Abdominal pain in 9 patients (52.9%) was relieved spontaneously.

In the long term, only the 96-year-old patient had mild abdominal pain after a few months of interruption, which was gradually relieved after oral administration of prednisone at 40 mg/day. The drug was tapered to withdrawal. The disease had no impact on the quality of life. Two patients (11.8%) died, including one due to respiratory failure caused by pneumonia and the other one because of pancreatic cancer with lung and liver metastases.

**Table 1:** Clinical characteristics and long-term outcomes in 17 mesenteric panniculitis patients

Patient	Sex	Age (years)	Neoplastic disease	Disease subtype	Gallbladder disease	Fever	Nausea	Vomiting	Predominant symptoms	Causes of abdominal pain	Medications	Long-term outcomes	
												Risk of recurrence	Impact of the disease on quality of life
A	Male	61	No	Fulminant	Gallstone	No	Yes	Yes	Severe abdominal pain	Acute pancreatitis, Mesenteric panniculitis	Antibiotics	No	No
B	Male	87	Pancreatic cancer	Chronic	Gallstone	Yes	No	No	Cachexia	/	Antibiotics	No	No
C	Male	43	No	Chronic	Gallbladder polyp	Yes	No	No	Mild abdominal pain	Mesenteric panniculitis	Antibiotics	No	No
D	Male	96	Melanoma	Chronic	Gallstone	Yes	No	No	Mild abdominal pain	Mesenteric panniculitis	Antibiotics, prednisone	Yes	No
E	Female	63	No	Fulminant	Acute cholecystitis, gallstone	Yes	Yes	Yes	Severe abdominal pain	Acute cholecystitis, Mesenteric panniculitis	Antibiotics	No	No
F	Female	64	No	Chronic	Gallstone	No	No	No	Mild abdominal pain	Mesenteric panniculitis	Antibiotics	No	No
G	Female	60	No	Chronic	No	Yes	Yes	Yes	Mild abdominal pain	Mesenteric panniculitis	Antibiotics + prednisone	No	No
H	Female	86	No	Chronic	Gallstone	Yes	Yes	Yes	Mild abdominal pain	Mesenteric panniculitis	Antibiotics	No	No
J	Male	86	No	Chronic	No	Yes	No	No	Mild abdominal pain	Mesenteric panniculitis	Observe	No	No
K	Male	72	No	Chronic	No	No	No	No	Mild abdominal pain	Mesenteric panniculitis	Observe	No	No
L	Male	52	Cardia cancer	Chronic	Gallstone	No	No	No	Cachexia	/	Observe	No	No
M	Male	37	No	Chronic	Chronic cholecystitis	No	No	No	Mild abdominal pain	Mesenteric panniculitis	Observe	No	No
N	Female	75	No	Chronic	No	No	No	No	Mild abdominal pain	Mesenteric panniculitis	Observe	No	No
O	Female	64	No	Chronic	Gallstone	No	No	No	Mild abdominal pain	Mesenteric panniculitis	Observe	No	No
P	Female	68	No	Chronic	Gallstone	No	No	No	Mild abdominal pain	Mesenteric panniculitis	Observe	No	No
Q	Female	65	No	Chronic	Chronic cholecystitis	No	No	No	Mild abdominal pain	Mesenteric panniculitis	Observe	No	No
R	Female	48	No	Chronic	No	No	No	No	Mild abdominal pain	Mesenteric panniculitis	Observe	No	No

## Discussion

The pathophysiology of MP remains unknown for the time being [13–15]. Typically, MP is also called “mesenteric lipodystrophy”, “mesenteric panniculitis”, “retractile mesenteritis” and “sclerosing mesenteritis”. The above terms mostly represent the characteristic histopathological variants featuring one typical mesenteric inflammation type with no involvement of vessel, lymph node and adjacent gut [15, 16]. It can be divided into three distinct stages [11, 14, 17]. In stage 1, also known as “mesenteric lipodystrophy”, mesenteric fat will be substituted by the foamy macrophages, and there was almost no inflammatory alterations on imaging findings [16]. It is usually asymptomatic. Stage 2, also referred to as “mesenteric panniculitis”, is characterized by inflammation, with infiltration of plasma cells, polymorphonuclear leukocytes, and foamy macrophages [16, 18]. Patients in this stage have abdominal pain, nausea and vomiting, fever and fatigue [19]. Stage 3, also called “retractile mesenteritis”, shows the typical feature of fibrosis, collagen deposition, as well as abdominal mass formation. The above-mentioned stages may co-exist or take place in a specific order, rather than separated from each other. Because serial biopsies were lacking, it was impossible to objectively establish the progression [20]. Pathological alterations in every stage involve the intestinal submucosal and mesenteric fats, which usually extend to submucosa and intestinal muscles. Intact mucosal structure is still observed [21]. In a series of studies on CT scans for a 5-year follow-up period, most patients (70.2–96.5%) had stable disease, 8–27% of them showed spontaneous resolution of symptoms, and a low proportion (2.3–16%) had disease progression [3, 4, 22–25].

The symptoms and signs of MP are nonspecific [20]. Typically, the most common complaint described in the literature is abdominal pain [1, 3, 16]. While there are many causes of abdominal pain. Therefore, it is necessary to judge the acute or chronic onset, location, nature and degree of abdominal pain in patients. When the medical history and physical examination are not sufficient to determine the pathological changes in the abdomen, especially in patients with acute abdomen of unknown etiology, CT examination can be considered [3]. CT scans vary depending on the severity of inflammation, and the findings observed include inflammatory soft tissue mass, pseudocapsule, ground-glass appearance of the mesenteric fat, fat ring sign, fatty haziness, and calcification caused by necrosis of adipose tissue [22]. Coulier [4] put forward the definite diagnosis of MP when 3 of 5 typical characteristics were satisfied: (sign 1) a well-defined “mass effect” on surrounding tissues without invasion; (sign 2) hyper-attenuation of mesenteric fat tissue compared with surrounding mesocolonic or retroperitoneal fat; (sign 3) presence of small soft tissue nodes (< 10 mm); (sign 4) the hypo-attenuation of fatty “halo sign”; and (sign 5) hyper-attenuation of pseudocapsule probably surrounding the whole entity. Notably, those last two signs may be inconstant yet quite specific.

Ultrasound examination has also been used in the diagnosis of MP, which probably reveal the well-defined hyper-attenuation of mesenteric mass with small hypo-attenuation of the center [26, 27]. Magnetic resonance imaging (MRI) shows one mesenteric mass that has a moderate signal intensity on T1 [28]. Whereas on T2-weighted images, the intensities can be different according to fibrosis (hypointensity) and edema (hyperintensity) levels [29]. Only a very few cases with ultrasound and MRI evaluation have been reported and standardized definitions are lacking.

Laboratory findings on MP are usually related to underlying inflammation; for instance, the incidence of leukocytosis is reported to be 16% [3, 18]. The erythrocyte sedimentation rate (ESR) and C-reactive protein (CRP) increased in 14–88% [18, 30], and low albumin is found in 5% of patients [18].

The diagnosis of MP is often made empirically based on the characteristic radiographic findings [31], pathology remains the gold standard. Biopsy is always performed in laparotomy or exploratory laparoscopy [32].

There is no guideline for the treatment of MP in the world [5, 33, 34]. MP is rare, which has added to the difficulty in assessing its therapeutic responses, leading to the empiric options. They are derived on the basis of individual cases, without using a specific method for determining symptom severity [34]. Corticosteroids, which are usually used as the initial therapy targeting the inflammatory component, show rapid and excellent responses. However, their long-term use is associated with certain complications such as hyperglycemia and peripheral neuropathy, which have limited their clinical application [5, 35]. Azathioprine, as the immunosuppressive agent with the longest history, has been widely used in the treatment of different diseases like inflammatory bowel disease and rheumatologic diseases [36, 37]. Bala et al. reported a case of MP with abdominal pain and bowel obstruction. The patient became disease free after 5-month treatment with steroid and azathioprine following partial ilectomy [36]. Cyclophosphamide is also effective on MP as an immunosuppressant. R W Bush et al. presented two patients with aggressive form of MP, which was characterized by a progressive, life-threatening course, prominent retroperitoneal lesions, and tubuloreticular structures in one case; besides, in both patients, cyclophosphamide resulted in prompt and dramatic symptom improvements, without disease recurrence [38]. Tamoxifen is a selective estrogen receptor modulator, which has exhibited benefits in patients with MP through the inhibition of fibroblast TGF-β1 production [39, 40]. As indicated in an open-label pilot study, thalidomide was effective on patients with symptomatic MP through exerting the anti-inflammatory properties [41]. Andreas N Kapsoritakis et al. described a 62-year old man with MP, who presented with pulmonary tuberculosis after initial therapy with corticosteroids. The patient was subsequently treated with pentoxifylline, with substantial clinical and radiological improvements [42]. Fasoulas et al. reported a case of MP in whom steroid dependence was successfully managed with colchicine without any recurrence [43]. R Mazure et al. described a patient with MP refractory to surgery who achieved a good response to oral progesterone (10 mg/day for 6 months) with complete disappearance of tumor mass and clinical symptoms [44], which might be associated with their fibrinolytic characteristics [45]. Some studies have also reported a good clinical response of MP to antibiotics, particularly for patients with elevated inflammatory markers [46, 47], similar to our results. In a small study involving 92 patients, it was reported that tamoxifen combined with prednisone led to significant symptomatic improvements in 60% of patients [30]. Surgery may be attempted if medical therapy fails or in the presence of life-threatening complications such as bowel obstruction or perforation [48]. Table 2 summarizes the different treatment options for MP, including the risks and benefits of each approach and their evidence level.

**Table 2:** Different treatment options for mesenteric panniculitis

Treatment options	Benefits	Risks	Evidence level
Corticosteroids	Anti-inflammatory	Long-term use is associated with some complications (e.g.,hyperglycemia and peripheral neuropathy)	IV
Azathioprine	Immunosuppressant in organ transplantation and autoimmune diseases	Myelosuppression, teratogenicity, abnormal liver function, and occasionally muscle atrophy	IV
Cyclophosphamide	Immunosuppressant and anti-tumor	Myelosuppression, urinary tract reactions, gastrointestinal symptoms, alopecia, stomatitis, toxic hepatitis, etc.	IV
Colchicine	Binding to neutrophil tubulin subunits alters membrane function, including inhibition of neutrophil chemotaxis, adhesion and phagocytosis	Gastrointestinal symptoms, muscle, peripheral neuropathy, myelosuppression, teratogenicity, shock, hair loss, rash, etc.	IV
Tamoxifen	An antagonist of the estrogen receptor in breast tissue via its active metabolite,4-hydroxytamoxifen	Vaginal dryness, discharge or irritation; reduced interest in sex and hot flashes	IV
Thalidomide	A sedative and an antiemetic agent. It has anti-inflammatory, immunomodulatory and antiangiogenic effects	Teratogenicity, lethargy, dizziness, rash, constipation, nausea, abdominal pain, facial puffiness, polyneuritis, allergies, etc.	IV
Pentoxyfylline	An antifibrotic agent	Nausea, dizziness, anorexia, bloating, etc.	IV
Progesterone	It downregulates proliferation and metabolism of fibroblasts and fibrogenesis	Occasionally nausea, dizziness, breast swelling, long-term application can reduce menstruation or amenorrhea, edema, etc.	IV
Antibiotics	Anti-infection	Drug resistance, anaphylaxis, double infection	IV
Surgical resection	Acute abdominal complications such as intestinal perforation, ischemia or obstruction requires urgent surgery	Trauma, a risk of surgical complications	IV

Typically, many patients may have abdominal pain, abdominal fullness, diarrhea, anorexia, nausea, weight loss or intestinal obstruction. MP patients with biliary dyskinesia are rare [49]. Due to the varying clinical presentations, it is challenging to make a definite diagnosis among these patients. Because of the close association of MP with malignancy [14, 35, 50–54], an initial evaluation that includes appropriate testing to rule out malignancies is a critical part of patient care. Therefore, a multidisciplinary discussion is needed. If MP is not diagnosed timely, worst outcomes and even death may occur in patients with acute abdominal complications such as intestinal perforation, ischemia or obstruction [5, 30, 55–59]. Occasionally, MP or its treatment can prove fatal [30, 60]. Prabin Sharma et al. conducted one systematic review on 192 MP patients, and they reported the frequent complications like bowel obstruction/ischemia (*n* = 10, 23.8%) or obstructive uropathy/renal failure (*n* = 10, 23.8%). Altogether 14 death cases were observed, including 12 (85.7%) dying of MP-associated complications [18]. Generally speaking, if MP patients respond well to the drugs, they have good prognosis [30].

## Conclusion

Little is known about the chronic inflammatory disorder MP. It accounts for a rare cause of non-classic abdominal symptoms. The etiology of MP is still unclear. Based on our report, prednisone appears to be effective. Therefore, clinicians should be aware of this disease and carefully solicit the history of one patient, since a simple denial can be misleading. More investigations are needed to shed more lights on MP and its optimum treatment.

## Data Availability

All data utilized and/or analysed in this work can be obtained from the corresponding author upon reasonable request.

## Abbreviations

MP: Mesenteric panniculitis
CT: computed tomography
COVID-19: Coronavirus Disease
CRP: C-reactive protein
ESR: erythrocyte sedimentation rate
